# A Survey for *Escherichia coli* Virulence Factors in Asymptomatic Free-Ranging Parrots

**DOI:** 10.5402/2012/984813

**Published:** 2012-07-10

**Authors:** André Becker Saidenberg, Neiva Maria Robaldo Guedes, Gláucia Helena Fernandes Seixas, Mariangela da Costa Allgayer, Erica Pacífico de Assis, Luis Fabio Silveira, Priscilla Anne Melville, Nilson Roberti Benites

**Affiliations:** ^1^Departamento de Medicina Veterinária Preventiva e Saúde Animal, Faculdade de Medicina Veterinária e Zootecnia, Universidade de São Paulo, 05508 270 São Paulo, SP, Brazil; ^2^Programa de MDR, Universidade Anhanguera-UNIDERP, and Projeto Arara-azul, 79051 660 Campo Grande, MS, Brazil; ^3^Projeto Papagaio-verdadeiro, Fundação Neotrópica do Brasil, 79290 000 Bonito, MS, Brazil; ^4^Universidade Luterana do Brasil, 92425 900 Canoas, RS, Brazil; ^5^Departamento de Zoologia, Instituto de Biociências, Universidade de São Paulo, 05508 090 São Paulo, SP, Brazil

## Abstract

Parrots in captivity are frequently affected by *Escherichia coli* (*E. coli*) infections. The objective of this study was to collect information on the carrier state for *E. coli* pathotypes in asymptomatic free-ranging parrots. Cloacal swabs were collected from nestlings of Hyacinth, Lear's macaws and Blue-fronted Amazon parrots and tested by polymerase chain reaction (PCR) for virulence factors commonly found in enteropathogenic, avian pathogenic, and uropathogenic *E. coli* strains. In total, 44 samples were cultured and *E. coli* isolates were yielded, from which DNA was extracted and processed by PCR. Genes commonly found in APEC isolates from Blue-fronted Amazon parrots and Hyacinth macaws were expressed in 14 of these 44 samples. One atypical EPEC isolate was obtained from a sample from Lear's macaw. The most commonly found gene was the increased serum survival (*iss*) gene. This is the first report, that describes such pathotypes in asymptomatic free-living parrots. The findings of this study suggest the presence of a stable host/parasite relationship at the time of the sampling brings a new understanding to the role that *E. coli* plays in captive and wild parrots. Such information can be used to improve husbandry protocols as well as help conservation efforts of free-living populations.

## 1. Introduction

Parrots are among the most endangered group of birds worldwide, and 15 of the 84 Brazilian species are classified as being vulnerable or critically endangered [1]. Studies on parrot populations are important to establish a database that can be assessed in the event of outbreaks, which could also be useful for subsequent epidemiological studies and conservation efforts [2]. However, health surveys of free-ranging wild animals are mostly focused on retrospective studies on mortality [3, 4]. Research on diseases affecting free-ranging parrots is still scarce and studies performed often fail to maximize the scientific information that could be gathered [2, 5–8]. Such data could be of extreme importance in guiding conservation measures *ex situ* and *in situ*.

The Hyacinth macaw and Lear's macaw are well-known flagship species that have suffered heavily owing to the destruction of habitat and illegal trade. A number of studies aimed at promoting their recovery have been performed and actions taken; however, they are still classified as endangered species and face severe threats to their long-term survival [9]. On the other hand, the Blue-fronted Amazon parrot is rated as a species of least concern regarding its conservation status [9]. It is, however, the most illegally traded parrot species in Brazil, and therefore, it is possible that decades of successive capturing of nestlings and concomitant ageing of the adult population could cause local extinctions in several areas where it is still common today [10].

Studies have established that the intestinal flora of most species of healthy captive psittacines is composed essentially of Gram-positive bacteria [11, 12]. Parrots in captivity are frequently affected by infections caused by Gram-negative bacteria, and these microorganisms are considered either pathogenic or opportunistic [13]. One such bacterium, *Escherichia coli *(*E. coli*), is frequently involved in respiratory, digestive, and septicemic disorders in captive parrots [13]. It is possible to classify *E. coli* into pathotypes by using genes responsible for the expression of virulence factors. Commonly described pathotypes include EPEC (enteropathogenic *E. coli*), APEC (avian pathogenic *E. coli*), and UPEC (uropathogenic *E. coli*) [14, 15]. EPEC is an important category of diarrheagenic *E. coli *and a major cause of infant diarrhea in developing countries, while APEC is recognized for significant economic losses to the poultry industry, resulting in respiratory diseases and septicemia [14, 15]. UPEC is a serious cause of urinary diseases in humans, causing cystitis that may progress to pyelonephritis [16]. Although, certain *E. coli* classification studies performed on wild birds [17–22] and those involving captive psittacines have found a certain degree of correlation between disease and specific pathotypes [23–25], current serological methods to determine the pathogenicity of *E. coli* strains do not accurately predict which strains will be pathogenic in which birds [13].

The purpose of this study was to test cloacal samples from asymptomatic free-living nestlings (Blue-fronted Amazon parrots as well as Hyacinth and Lear's macaws) to determine if they could be carriers of recognized *E. coli *pathotypes. In addition, we discuss the role that these strains could play in both free-living and captive parrots.

## 2. Materials and Methods

### 2.1. Microbiological Examination

The samples of this study were collected during field surveys of the nestlings of Hyacinth macaws and Blue-fronted Amazon parrots in the Pantanal (wetlands) region of the Refúgio Ecológico Caiman and neighboring farms (19°58′S, 56°24′W) in Mato Grosso do Sul State, and of Lear's macaws at the Estação Ecológica de Canudos (09°53′48′′S, 39°01′35′′W) in the Caatinga (semi-arid) of the Bahia State. Cloacal swabs (CultureSwab Sterile, DIFCO Becton Dickenson and Company, Sparks, Maryland, USA) were moistened with care using a sterile saline solution so as not to contaminate the swab during insertion in the cloaca. All chicks had no evident signs of disease (soiled vent, emaciation, prostration, or delays in development according to their estimated age). In total, 44 samples were obtained, of which 10 were from Hyacinths, 13 from Lear's, and 21 from Blue-fronted Amazon parrots.

The swabs were refrigerated up to processing at which point they were aerobically incubated in BHI broth (Brain Heart Infusion, DIFCO) for 24 hours at 37°C. They were then streaked onto MacConkey (DIFCO) agar plates and incubated for another 24 hours at 37°C. Bacteria were identified using a specific enterobacteria identification kit (Newprov, Pinhais, Paraná, Brazil) and stored at −20°C. Isolates were tested using polymerase chain reaction (PCR) (Table 2.), for the presence of *E. coli* attaching and effacing (*eae*) gene and bundle-forming pili structural (*bfp*A) gene of EPEC [26]. For APEC, the aerobactin (*iuc*D), cytotoxic necrotizing factor (*cnf*1), S fimbrial adhesin (*sfa*), and P fimbrial adhesin (*pap*EF) genes were amplified. UPEC utilized alpha hemolysin (*Hly*A) in addition to the genes used for APEC [27]. Additional APEC genes for serum resistance (*iss*) and temperature-sensitive hemagglutinin (*tsh*) were also tested [28].

## 3. Results

*E. coli *was obtained from all 44 samples. Details on the positive samples are given in Table 1. One sample was positive solely for *eae* and not for *bfp *and was characterized as atypical EPEC. This sample was collected from a Lear's macaw chick. A large number of samples were positive for virulence factors commonly found in APEC (14 samples), with most originating from Blue-fronted Amazon chicks. The gene most frequently found was *iss *(14 positive samples). This gene was also found associated with other virulence factors in 4 samples, all from Blue-fronted Amazon parrots. There was also an association between other virulence factors among some isolates (Table 1).

## 4. Discussion

EPEC have the ability to cause lesions on the intestinal mucosa, leading to severe diarrhea. This process is initiated by adherence to the epithelial cell membrane and is mediated by the adhesin intimin (encoded by the *eae* gene) [14]. Typical EPEC strains possess both intimin and bundle-forming pili (encoded by the *bfp* gene), which are responsible for the initial contact between the bacteria and the host cell; atypical isolates, on the other hand, lack the *bfp* gene [29]. Humans are considered the primary reservoir for the typical pathotype although it has also been found in dogs and cats [29, 30].

One of the samples in this study was characterized as an atypical EPEC. These strains are frequently isolated from domestic animals. There are few reports of the presence of atypical EPEC in birds, especially the isolates obtained from poultry [30–32]. There are also reports of atypical EPEC causing fatal outbreaks in backyard passerine species [18], and carriers have been found among feral pigeons and rehabilitated seagulls [20, 22]. A study using 103 samples from captive psittacines detected 4 samples characterized as typical EPEC and 3 others as atypical isolates, all of which originated from clinical cases of diarrhea, enteritis, or septicemia [23]. Another survey conducted in Brazil regarding necropsy cases of symptomatic parrots also showed the presence of the *eae* gene in 2 atypical samples of *E. coli* isolated from the livers of 2 individual pet Amazon parrots [24]. The fact that we found an atypical isolate in an asymptomatic bird, especially a free-ranging individual, suggests that this pathotype is not pathogenic in all parrots depending on the situation to which the birds are subjected.

APEC and UPEC share several genes that encode virulence factors such as P fimbrial adhesin (*pap* gene) and S fimbrial adhesin (*sfa* gene) [33]. These genes allow UPEC to bind and invade host cells and tissues [34]. APEC has been extensively studied in poultry where it has been observed that the P fimbrial adhesion enables binding of bacteria to internal organs and protects against heterophilic inflammation [35], while the S fimbrial adhesin is associated with omphalitis, salpingitis, chronic respiratory diseases, and sepsis [36]. Although several virulence factors have been associated with clinical cases of APEC in poultry, no specific factor has been confirmed to be responsible for contributing to the pathogenicity observed [37], which makes it difficult to interpret results among lesser-studied groups such as wild birds.

Siderophores, such as aerobactin (*iuc* gene), enable *E. coli* to obtain iron stores from the host, and strains with this gene are quite frequently associated with clinical cases of poultry [38]. One sample isolated from a Blue-fronted Amazon was positive for this gene. Toxins, such as alpha hemolysin (*hly*A gene—characteristic of UPEC) and cytotoxic necrotizing factor 1 (*cnf*1 gene), provide the ability to cause tissue damage, contributing to dissemination and release of host nutrients while impairing the immune defenses [34].

In this investigation, 2 samples were positive for the *sfa* and 1 was positive for the *cnf*1 gene. These genes have been reported in pathogenic strains of *E. coli* isolated from septicemic poultry [36], as well as from human and domestic animals with extraintestinal pathogenic *E. coli* infections [16, 34]. A survey dealing with healthy feral pigeons detected a number of positive cloacal-swab specimens for the cytotoxic necrotizing factor 1, showing the potential for disease spread by carriers of this species [22].

Other genes such as the *tsh *(temperature-sensitive hemagglutinin) and the *iss* (increased serum survival) are reported to be present in APEC strains. The increased serum survival causes sepsis by conferring resistance to the bacteria against the host immune bactericidal defenses [37]. The exact function of *tsh *is largely unknown, but it has been shown to be involved in mechanisms of adherence to the respiratory tract of poultry [39]. In this study, 6 samples were positive for the *iss* gene. However, it has been shown in poultry that the presence of the *iss* gene alone may not be sufficient to identify APEC isolates because this gene can also be found in the intestinal microbiota of healthy individuals, and an association with the *iuc* gene was reported to be necessary for achieving higher levels of virulence [37]. Interestingly, a sample from an asymptomatic Blue-fronted Amazon chick in this study showed an association between the *iss* and *iuc* genes.

This research also found 1 positive sample for the *iss*/*tsh* association. In domestic turkeys, a relationship between clinical cases of colibacillosis and the presence of *iss *and *tsh *has been described [40]. The occurrence of gene associations such as *iss/pap*, *iss/iuc/tsh*, and *pap/iuc/tsh *has previously been reported in *E. coli *isolated from fecal, liver, and blood samples collected during necropsies performed in symptomatic psittacines in Brazil. These results in captive birds demonstrate that there is a connection between the presence of these genes and some clinical cases of colibacillosis as a contributing cause of death whether as primary or opportunistic pathogens [24].

Even though the sample numbers were too small to reach definite conclusions, we observed differences in the presence of virulence factors among the different species. APEC/UPEC genes were found mostly in species that usually nest on trees and live in a tropical climate (at least in this studied area, for the species *A. hyacinthinus* and *A. aestiva*). EPEC was found in species that nest on limestone cliffs and inhabit semi-arid regions (*A. leari*). Differences among feeding habits, direct or indirect contact with other wild animals, and human activities (interference due to human settlements and domestic animals) could possibly have influenced these results, and thus, these results should be further investigated. 

Although previous studies showed that some virulence factors are indeed involved in clinical cases of colibacillosis in psittacines [23–25], this investigation found a number of carriers for virulence factors. These unusual findings focus the attention on the fact that, at least at the time of sampling, there was a stable host/parasite relationship. Unlike wild birds, parrots maintained in captivity are frequently exposed to a number of factors that cause immunosuppression and increase their susceptibility to disease. These include deficient diets, inadequate hygiene, and lack of mental and physical stimuli; all of these are factors that may determine the course of the disease when the animal is exposed to a microorganism [41].

The concept of disease is considered the result of an interactive relationship among the causative agent, the animal, and environmental factors [42], and a multitude of factors act together in order to initiate the disease process. If a factor is not present, it is probable that the organism will be capable of fighting the pathogen without showing overt clinical signs. The nestlings in our study successfully fledged, indicating that although the potential for disease was present, birds living in their natural environment, without the factors induced by captivity, are more likely to remain disease free.

The results presented here are also important for the future conservation of the 2 endangered species (*A. hyacinthinus* and *A. leari*) as well as the heavily trafficked *A. aestiva* because they could better guide *ex situ *husbandry practices involved in captive breeding and rehabilitation/relocation programs, besides assisting monitoring of the overall health of the wild population.

In conclusion, to our knowledge, this is the first study that tested *E. coli* virulence factors in wild psittacines. It is also the first to describe *E. coli *carriers in free-ranging parrots, and the results indicate that although the potential to develop disease was present, several factors that are most likely to be found in captivity needed to be involved in triggering disease development. Other studies involving different species as well as a higher number of samples are important to further define the role and risks involved with specific *E. coli *pathotypes in the case of both wild and captive psittacines.

## Figures and Tables

**Table 1:** Cloacal swab samples from free-ranging parrot nestlings tested for select *Escherichia coli* virulence factors. Surveyed species and the number of positive samples for each tested gene/association by polymerase chain reaction (PCR) are shown.

Species	Number of positive samples/total number of samples	*eae*	*bfp*	*sfa*	*pap*	*iss*	*iuc*	*tsh*	*HlyA*	*cnf1*
*Amazona aestiva*	7/21	−	−	−	−	+	−	−	−	−
*Amazona aestiva*	1/21	−	−	+	−	+	−	−	−	−
*Amazona aestiva*	1/21	−	−	−	−	+	−	+	−	−
*Amazona aestiva*	1/21	−	−	−	−	+	+	−	−	−
*Amazona aestiva*	1/21	−	−	+	−	+	−	−	−	+
*Anodorhynchus hyacinthinus*	3/10	−	−	−	−	+	−	−	−	−
*Anodorhynchus leari*	1/13	+	−	−	−	−	−	−	−	−

**Table 2:** Virulence factors and gene sequences tested by polymerase chain reaction (PCR) according to the described references.

Virulence factor	Sequence (5^′^–3^′^)	Reference
Intimin *(eae) *	F-CTGAACGGCGATTACGCGAA	Aranda et al. [26]
	R-CGAGACGATACGATCCAG	
Bundle forming pili *(bfp) *	F-AATGGTGCTTGCGCTTGCTGC	Aranda et al. [26]
	R-GCCGCTTTATCCAACCTGGTA	
S fimbrial adhesin *(sfa) *	F-CTCCGGAGAACTGGGTGCATCTTAC	Yamamoto et al. [27]
	R-CGGAGGAGTAAT TACAAACCT GGCA	
P fimbrial adhesin *(pap) *	F-GCAACAGCAACGCTGGTTGCATCAT	Yamamoto et al. [27]
	R-AGAGAGAGCCACTCTTATACGGACA	
Increased serum survival *(iss) *	F-AGAGAGAGCCACTCTTATACGGACA	Ewers et al. [28]
	R-CTATTGTGAGCAATATACA	
Aerobactin *(iuc) *	F-TACCGGATTGTCATATGCAGACCGT	Yamamoto et al. [27]
	R-AATATCTTCCTCCAGTCCGGAGAAG	
Temperature-sensitive hemagglutinin *(tsh) *	F-ACTATTCTCTGCAGGAAGTC	Ewers et al. [28]
	R-CTTCCGATGTTCTGAACGT	
Alpha hemolysin *(hlyA) *	F-AACAAGGATAAGCACTGTTCTGGCT	Yamamoto et al. [27]
	R-ACCATATAAGCGGTCATTCCCGTCA	
Cytotoxic necrotizing factor *(cnf1) *	F-AAGATGGAGTTTCCTATGCAGGAG	Yamamoto et al. [27]
	R-CATTCAGAGTCCTGCCCTCATTATT	
